# Curcumin, Curcumin Nanoparticles and Curcumin Nanospheres: A Review on Their Pharmacodynamics Based on Monogastric Farm Animal, Poultry and Fish Nutrition

**DOI:** 10.3390/pharmaceutics12050447

**Published:** 2020-05-11

**Authors:** Mohammad Moniruzzaman, Taesun Min

**Affiliations:** Department of Animal Biotechnology, Jeju International Animal Research Center (JIA) & Sustainable Agriculture Research Institute (SARI), Jeju National University, Jeju 63243, Korea; mzaman_bfri@yahoo.com

**Keywords:** curcumin, nanoparticle, nanospheres, monogastric animal, poultry, fish, nutrition

## Abstract

Nanotechnology is an emerging field of science that is widely used in medical sciences. However, it has limited uses in monogastric farm animal as well as fish and poultry nutrition. There are some works that have been done on curcumin and curcumin nanoparticles as pharmaceutics in animal nutrition. However, studies have shown that ingestion of curcumin or curcumin nanoparticles does not benefit the animal health much due to their lower bioavailability, which may result because of low absorption, quick metabolism and speedy elimination of curcumin from the animal body. For these reasons, advanced formulations of curcumin are needed. Curcumin nanospheres is a newly evolved field of nanobiotechnology which may have beneficial effects in terms of growth increment, anti-microbial, anti-inflammatory and neuroprotective effects on animal and fish health by means of nanosphere forms that are biodegradable and biocompatible. Thus, this review aims to highlight the potential application of curcumin, curcumin nanoparticles and curcumin nanospheres in the field of monogastric farm animal, poultry and fish nutrition. We do believe that the review provides the perceptual vision for the future development of curcumin, curcumin nanoparticles and curcumin nanospheres and their applications in monogastric farm animal, poultry and fish nutrition.

## 1. Introduction

Biotechnology and nanotechnology are considered as the 21st century’s most emerging and advanced technologies. The term nanotechnology was first coined by Norio Taniguchi in 1974 [1]. Nanotechnology (from the Latin nanus, meaning the dwarf) can be defined as the understanding, design, development and application of materials and devices whose least functional makeup is on a nanometer scale (~1 to 100 nm) or one billionth of a meter (10^−9^) [2]. In brief, nanotechnology deals with the conversion of larger molecules to nanometer size and it changes the physico-chemical nature of the cell matrix in terms of human or animal welfare. The changes may occur based on solubility, absorption, the transporting system, excretion and antagonistic behavior of the nanomaterials [3]. Nanobiotechnology can be an indispensable tool to monitor the individual cell development at the individual molecule level [4]. Nevertheless, one of the most important and extensive uses of nanobiotechnology is the area of human medicine [5], where it provides nanomaterials with the controlled drug delivery system for cancer remediation [6], nutrient utilization [7], hormonal controls [8] and gene therapy [9]. In case of animal welfare, the technology can be utilized to improve growth [10,11] and meat quality [11], reproductive performance [12,13], immunity enhancement [14] as well as disease resistance [10], antioxidant activity [15,16,17], and intestinal function [16] including food preservation [18]. Nanomaterials can be classified into four categories: metals, polymers, natural compounds and nanostructured materials [19]. Metals are usually mineral nanoparticles such as gold [20], copper [21], selenium [22], palladium [23] etc. However, the main problem of using metal nanomaterial is its non-biodegradability [19]. Polymers of nanomaterials are pieces of nanometer-sized polymers which are biodegradable and biocompatible and can be utilized very well [24]. The biocompatibility is the most important concern in nanobiotechnology in order to have low or no toxic effects on the organism [25,26,27,28]. Nanoparticles that come from natural sources are known as natural compounds (natural polymers or proteins) which are highly biodegradable, biocompatible and distributable in the body [19]. Nanostructured materials are the conjugations of lipid or protein with nanoparticles such as phospholipid-based nanoparticles (curcumin nanospheres) or phosphoprotein-based nanoparticles (casein micelles). From a nutritional point of view, natural and nanostructured nanoparticles have great advantages in terms of nutrient delivery by means of encapsulation or adhesion of the nanomaterials. However, natural or nanostructured nanoparticles may be toxic in high doses if they are not properly adjusted in the organisms. In animal nutrition, nanotechnology has been discussed regarding the nature of food and its taste, texture, processing time, thermal susceptibility, stability, safety and bioavailability of nutrients as well as the feeding of animals [29]. Nutrients can be delivered in the form of nanoparticles or in aided forms such as nanocapsules and emulsifications as well as nanosphere forms.

In recent years, phytogenic natural compounds are widely used in animal feeds as antimicrobial growth promoters (AGPs) to replace the antibiotics in monogastric animal [30,31], poultry [11] or aquaculture [32] feed since the indiscriminate use of antibiotics in animal feed may favor the emergence of antibiotic-resistant strains of bacteria [11] and their accumulation in edible tissues [33]. In fact, the European Union (EU) has already banned AGPs in animal feeds, and the Food and Drug Administration (FDA) of the USA is going to restrict the use of AGPs in animal feeds [34,35]. For animal disease resistance and growth enhancement, currently researchers are putting emphasis on the use of natural growth promoters and non-antibiotic growth promoters (NGPs) in terms of improvement of the gastrointestinal tract (GIT) through the incorporation of various feed additives, for example, curcumin and its derivatives which have antimicrobial, antioxidant, anti-inflammatory, appetite increasing, immune-modulatory and gastroprotectve effects on animal health [11].

Turmeric (*Curcuma longa* L.), a tropical rhizomatous herb of the *Zingiberaceae* family, is regarded as the “golden spice”, used as curry powder in South and South-East Asian cuisine [11,36]. Turmeric has a medicinal value in human and animal health [37]. The dried turmeric rhizome consists of 3–6% terpenes and terpenoids, 6–8% protein, 6–10% fat, 60–70% carbohydrate and 3–6% fiber [38]. Curcumin or diferuloylmethane [1,7-bis(4-hydroxy-3-methoxyphenyl)-1,6-heptadiene-3,5-dione] is a hydrophobic and polyphenolic compound extracted from the perennial herb, *Curcuma longa* [39,40,41]. Curcumin, is a yellow pigment, found in turmeric at 3–6% with bioactive properties [37]. The melting point of curcumin ranges from 176 to 177 °C [42]. Commercial curcumin usually has 77% diferuloylmethane, 17% dimethoxy-curcumin and 6% bisdemethoxycurcumin [43]. Curcumin has a relatively low toxicity and is generally regarded as safe (GRAS) which defined as the chemical or substance added to food is considered as safe by experts, and so is exempted from the usual Federal Food, Drug, and Cosmetics Act food additive tolerance requirements by the United States Food and Drug Administration (FDA) [44]. For many years, curcumin has been used in Indian ayurvedic remedies and Chinese medicines [44,45]. Despite the beneficial effects of curcumin, it has some limitations such as low water solubility (hydrophobic), an unstable chemical structure, being rapidly metabolized but poorly absorbed in the body as well as its utilization or bioavailability differing depending on the species and sex [41]. Likewise, Wahlstrom and Blennow [46] reported that oral administration of curcumin (1 g/kg) to rats resulted in a huge excretion of curcumin (about 75%) in the feces with a small amount in the urine and blood plasma. However, Patil et al. [47] postulated that the piperine in black pepper could enhance the bioavailability of curcumin by 20-fold and CYP3A4 in cytochrome P450, p-Glycoprotein and uridine diphosphate (UDP)-glucuronosyltransferase (UGT), the enzyme responsible for glucuronosylation, increased the solubility of curcumin. 

Curcumin in the form of a nanoparticle (hereafter, curcumin nanoparticle) is a widely reported form for enhancing the bioavailability and solubility of lipophilic curcumin [10,48]. Kurita and Makino [49] as well as Hani and Shivakumar [50] reported that the solubility and absorption rate of nanocurcumin is higher than the normal curcumin form, respectively. Furthermore, curcumin nanoparticles can be more bioavailable and deposited more highly than the normal curcumin in comparison of the tissues of the Sprague-Dawley rat model [51,52]. However, no form of curcumin or its closely related analogues poses the properties required for a good drug or additive candidate in terms of chemical stability, high water solubility, potent and selective target activity, high bioavailability, broad tissue distribution, stable metabolism and low toxicity [53].

Due to the problems of water insolubility and low bioavailability of curcumin or curcumin nanoparticles, it has been reported that biodistribution and bioavailability of curcumin or curcumin nanoparticles would be increased by encapsulation processes such as nanoemulsions, liposomes, micelles, polymeric micro or nanoparticles, phospholipid complexes and hydrogels which showed the potential for efficient drug delivery systems that minimize the delay and reduce the vulnerability of diseases in organisms [44,54,55]. Prasad et al. [37] postulated that an increased amount and length of curcumin in blood circulation could enhance the tissue deposition and biological activity in animals. Furthermore, Mohamed et al. [56] opined that hydrogen-bromide-treated curcumin can be exclusively used as a source of antioxidant in bioactive food materials. As an organic curcumin nanocarrier, liposomal curcumin is considered as the best way of improving bioavailability of curcumin in cellular level [57] and the commodities based on liposomal formulations are being marketed for different dietary applications of curcumin [58]. In addition, in case of inorganic nano formulations, mesoporous silica nanoparticles (MSN) are the most used nanosystems for improving the bioavailability of poorly water soluble drugs [59,60,61,62]. 

**Figure 1:**
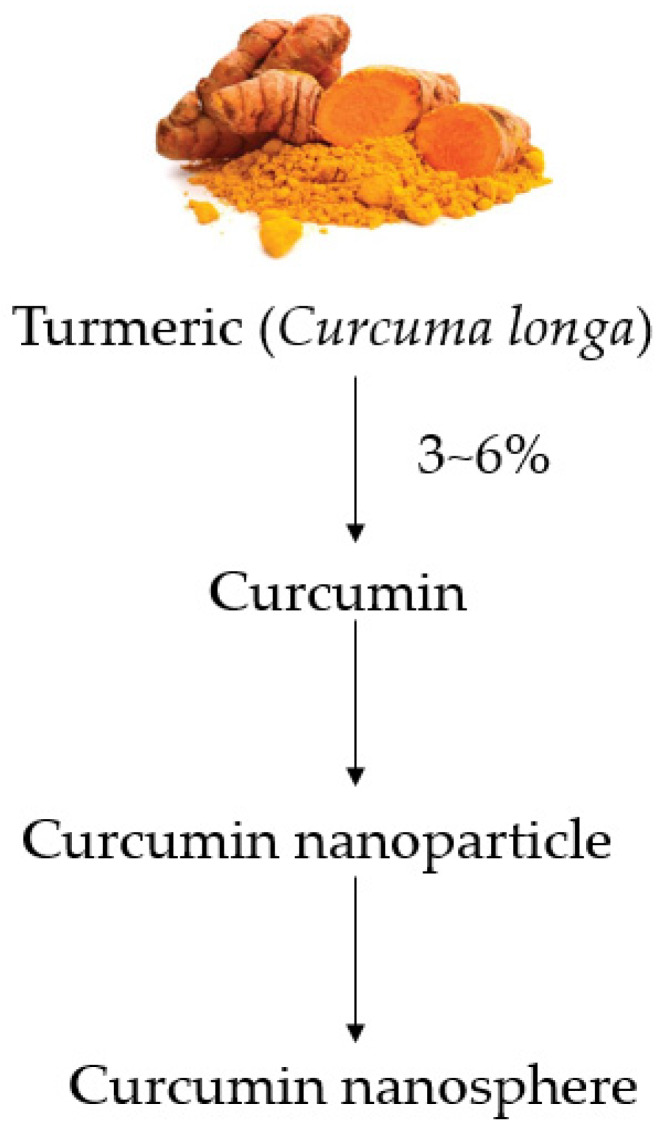
Outline of the production process from turmeric to curcumin nanospheres.

Recently, there are a significant number of research works have been conducted on curcumin or curcumin nanoparticles alone or in combination with other additives in the diet of monogastric farm animals and poultry as well as fish (Figure 1) [14,31,63,64,65,66,67,68,69]. Most of the studies dealt with growth enhancement, improvement of reproductive health and metabolism, antioxidants and immunomodulatory effects of curcumin or curcumin nanoparticles. However, a scarce number of articles were reported regarding the nanospheric forms [70,71]. This review article discuss the recent advancement of curcumin research based on nutritional studies in monogastric animals such as pig, poultry and fish. We hope that the review article will benefit the scientific communities for the further development of curcumin research in nutritional science. 

## 2. Curcumin or Nanocurcumin in Monogastric Farm Animal, Poultry and Fish Nutrition

Indiscriminate use of antibiotics in monogastric farm animal and poultry farming as well as in aquaculture has caused the emergence of new pathogenic strains. These consequences have warranted the development of safe and alternative growth promoters and immunity enhancers in farm animal, poultry and fish production. Monogastric farm animals like swine, poultry and fish are usually reared in intensive production systems which may ultimately affect their health status through different types of diseases and secondary infections and ultimately reduce their production performance [72,73,74,75,76]. Use of phytogenic additives in animal, bird and fish feed is a centuries-old practice. With vast potential areas of application, nanotechnology is still in its infancy for animal feed and nutritional studies [4]. The most common health-enhancing prophylaxis is nutritional strengthening of the gastrointestinal system in animals [77]. In this section we discuss the use of curcumin or turmeric (as the source of curcumin), and nanocurcumin based on the nutritional aspects related to the growth, reproductive capacity, digestibility, stress response, immune functions and histopathology as well as disease distance in different age stages of swine, rabbit, poultry and fish (Table 1, Table 2, Table 3 and Table 4). 

### 2.1. Dietary Turmeric/Curcumin in Swine Nutrition

In swine, the weanling period is the most crucial time from a nutritional perspective because at this stage young piglets are quickly shifted from sow’s milk to a dry- and low-digestible-starch-based diet which may ultimately affect their digestive and immune systems and finally cause morbidity or mortality in pigs [78]. The consequences may be due to distinctive changes in gastrointestinal tract (GIT) architecture, changes in autochthonous bacteria in the gut which deteriorate the small-intestinal barrier function and absorption of potential nutrients [79,80]. In the weanling period, low feed intake and feed efficiency, body weight loss and diarrhea are common phenomena in piglets. In farm levels, nearly 50% growth retardation may occur at the weaning stage of pigs [80]. To overcome this problem and to increase the post-weaning growth of pigs, farmers usually depend on some commercial antibiotics in the diets of the weanling pigs. However, the indiscriminate use of therapeutic antibiotics in farm animals may create the development of antibiotic-resistant strains of bacteria which may be harmful for the animal as well as the consumers in turn. Different countries of the world are now thinking to minimize or completely eliminate the inclusion of antibiotics in the diet for farm animals. To increase the production and health status of pigs, it is imperative to find out the alternatives to antibiotics which can be used either singly or in combination that are able to overcome stunted growth as well as the problems in the digestive system of weanling pigs. Different dietary components depending on their solubility, digestibility, viscous-forming ability and acid-buffering ability can potentiate or stimulate the gut health of pigs by preventing proliferation of pathogenic bacteria [78]. In this regard, dietary turmeric or curcumin have shown their positive effects in swine nutrition [31,68,69,81,82,83,84,85,86]. Maneewan et al. [81] reported that dietary turmeric can induce nutrient digestibility, blood parameters and intestinal gut health in nursery piglets when they offered 0.10% and 0.20% turmeric in the diets. In another reports, Liu [82,83] postulated that dietary turmeric oleoresin or in combination with capsicum oleoresin and garlic botanical could enhance the weight gain and gut mucosa health by alleviating diarrhea and reducing inflammation caused by *E. coli* bacteria. Dietary curcumin extracted from the turmeric rhizome also proved its promising effects as an immune stimulant in weaned piglets. As an antibiotic replacer and potential additive, dietary curcumin at 300 or 400 mg/kg of diet has shown its effectiveness in replacing quinocetone by means of enhancing growth performance, jejunal mucosal barrier integrity in the intestine and stimulating the immune system of weaned piglets challenged with enterotoxigenic *E. coli* [31]. But, Ilsley et al. [84] reported that dietary curcumin (200 mg/kg) had no effect on the growth and immune system of weaned piglets in relation to quillaja saponin. Intrauterine growth retardation (IUGR) which is defined as the growth below the 10th percentile for gestational age, has adverse effects on animals as demonstrated in pigs in terms of stunted growth, fatty liver and insulin resistance [68,69]. However, these conditions can be attenuated by the supplementation of curcumin in the diets of IUGR piglets. In addition, dietary supplementation of curcumin at 200 mg/kg can alleviate the jejunum damage through the Nrf2/Keap1 pathway in the intestine of IUGR growing pigs. Curcumin with other phytogenic additives such as resveratrol has shown combined effects in replacing dietary antibiotics in pigs. Resveratrol, like curcumin, has potential anti-inflammatory, bacteria-regulating and immune-promoting effects [85]. In a study, dietary curcumin (300 mg/kg) in combination with resveratrol (300 mg/kg) showed regulatory effects on gut microbiota, down-regulating the Toll-like-receptor 4 mRNA (TLR4) signaling pathway, reducing the intestinal pathology and enhancing the immunity by secreting immunoglobulin in weaned piglets. 

Piglets are often subjected to long road transportation. This consequence may cause different physical problems such as very high or low temperature, motion, long fasting, dehydration as well as a crowding problem. Dietary curcumin has shown neurological effects during pigs’ transportation. Wei et al. [86] postulated that dietary curcumin can increase the brain hippocampal nitric oxide (NO) production and reduce brain-derived neurotrophic factor (BDNF) gene expression which in turn could protect from subacute stress in pigs.

### 2.2. Dietary Turmeric/Curcumin in Poultry Nutrition

As the world human population is increasing, the demand for poultry meat also increasing. In poultry industry, birds are usually raise in extremely crowded condition which causes poor hygiene and stunted growth in chickens. To solve this problem, the antibiotics are extensively use to enhance chicks growth and health status. Due to the abuse of antibiotics and antimicrobial resistance matter, some phytogenic compounds especially turmeric or curcumin could be used to reduce the overwhelming dependence on antibiotics as well as increase the production performance of poultry industry [73]. A numerous studies have been conducted on the efficacy of turmeric or curcumin in poultry nutrition. In poultry industry, necrotic enteritis (NE) and avian coccidiosis are commons among the most important infectious diseases which resulted extensive mortality and substantive economic losses world-wide [87]. In a study, Lee et al. [87] demonstrated that dietary supplementation of turmeric oleoresin (4 mg/kg) and *capsicum* oleoresin (4 mg/kg) extracts could enhance the growth performance, reduce the gut lesions as well as serum levels of *Clostridium perfringens* (etiological agent of NE) toxins which resulted from molecular and cellular immune changes and finally showed their resistance against avian NE. In addition, dietary turmeric powder (TP) in single (7 g/kg) or in combination with neem and vitamin E have shown to increase growth, hematology, body composition, meat quality and serum biochemical parameters in broiler chicken [88,89]. However, Qasem et al. [90] reported that dietary turmeric powder (20 g/kg) did not improve the growth and immunity in broiler chickens. In case of Wenchang broiler chickens, dietary turmeric extract (100 to 300 mg/kg) could enhance the growth performance, breast muscle content and reduce the fat content in abdomen of chicks [91]. Moreover, Akhavan-Salamat et al. [92] demonstrated that dietary turmeric rhizome powder (TRP) or betaine (Bet) alone or in combination could improve the hematology and immune responses but TRP is more effective than Bet in terms of tolerance to heat stress in male Ross chicks. In laying hens, dietary TP showed no effects on body or egg weight and production at higher level (4% TP/kg), however, low level of TP (2% TP/kg) could increase egg production and quality slightly in Hisex laying hens [93]. Mirbod et al. [94] reported that increasing dietary turmeric or *Curcuma longa* rhizome powder (CRP) could enhance the egg quality in terms of improving egg shell thickness and hardness but abating cholesterol level in egg yolk in Leghorn laying hens. Nowadays, quail raising is getting much attention to meet the protein demand in addition to chickens. Dietary turmeric has shown its positive effects in quail rearing [12,95,96]. Turmeric (0.5%) with exogenous commercial enzyme, Panzyme (0.1%) has proven to increase growth performance and immune status in Japanese quail. Furthermore, dietary supplementation of turmeric in parent stock of Japanese quail may increase the egg quality in terms of higher vitellogenin, high density lipoprotein (HDL), vitamin A, B12, white egg protein and decrease the low density lipoprotein (LDL), total fat contents in eggs, in addition, increase the carcass content and antioxidant activity in female offsprings.

Nevertheless, curcumin extracted from turmeric has beneficial effects on poultry nutrition. Curcumin and turmerones extracted from lipophilic turmeric have shown their combined effect on the increment of growth, antioxidant and antimicrobial activities in broiler chickens [11]. Kim et al. [97] reported that dietary curcumin can be coccidiosis resistant challenged with *Eimeria* by means increasing body weight, and reducing fecal oocysts, gut lesions as well as increasing body immunity in Ross broiler chickens. On the other hand, Galli et al. [14,66] demonstrated curcumin (30 and 50 mg/kg) alone can reduce the lipid peroxidation and increase the antioxidant level in eggs of laying hens or curcumin combined with microencapsulated phytogenics (carvacrol, thymol and cinnamaldehyde improve the growth, flesh quality and unsaturated fatty acids in broiler chicks. Curcuminoids (222 mg/kg) in feeds showed protective effects on aflatoxin B1 (1 mg/kg) toxicity in male Ross broiler chicks [15] or curcuminoids (60 mg/kg) with tuna oil could increase the breast fillet and inhibit the auto-oxidation in thigh meat in slow growing chickens [98]. Rajput et al. [99,100] reported that supplementation of curcumin at 200 mg/kg can improve the growth, fat metabolism and intestinal integrity, whereas; curcumin (150 mg/kg) with lutein (150 mg/kg) could enhance the freshness of meat and antioxidant capacity in Arbor Acres broiler chickens. In addition, dietary curcumin alone (50 and 100 mg/kg) could improve the meat quality and oxidant stability in muscle as well as activate the glutathione (GSH) related enzymes and detoxify the Nrf2-mediated phase II detoxifying enzyme systems in broiler chickens [17,101,102]. Xie et al. [65] explained that curcumin has important role in reducing fat deposition in abdomen by decreasing liver and blood lipid contents, and lipogenic gene expressions. Thermal or temperature and feed-borne aflatoxin B1 (AFB1) effects are the most crucial events in poultry industry. The supplementation of dietary curcumin can attenuate the AFB1 toxicity and resistance to heat stress in broiler and laying hens in terms of different attributes such as growth increment, as well as inhibition of hepatoxicity, CYP2A6 enzyme-mediated bioactivation of AFB1, enhancement antioxidant biomarkers, blood chemistry and immune related gene expressions [103,104,105,106]. In case of Japanese quail, Sahin et al. [107] reported that high ambient temperature (34 °C) causes oxidative stress in quail, however, dietary curcumin supplementation (200 or 400 mg/kg) could ameliorate the problem by increasing body weight, feed intake and reducing serum malondialdehyde (MDA) and heat shock protein 70 (HSP70) as well as modulating Nrf2/HO-1 pathway in quail. Ruan et al. [16,108] postulated that supplementation of curcumin (200 to 800 mg/kg) could enhance the growth, serum GSH, jujunal mucosa and villus surface in intestine and reduce MDA, interleukin-1β, tumor necrosis factor-α as well as detoxify ochratoxin A-related gene expressions in ducklings. 

Use of curcumin nanoparticle or nanocurcumin in animal nutrition is a very recent event. In poultry nutrition, Rahmani et al. [10,109] reported that male broiler chicks (Ross 308) had decreased weight gain and high feed conversion ratio, and increased blood partial pressure of carbon dioxide (pCO_2_) and hematocrit at cold temperature (13–15 °C), however, when the chicks were offered feed with curcumin or nanocurcumin, the body weight and villus surface in intestine increased but MDA and caecal *Escherichia coli* decreased in broiler chicken at cold stressed condition. In another report, Marchiori et al. [12] demonstrated that thermal stress in quail negatively affects performance, however, dietary curcumin (30 mg/kg) and nanoencapsulated curcumin (10 mg/kg) could effectively enhance the quail health in terms of performance production, egg quality like antioxidant capacity of egg yolk, reduce feed conversion, lower thiobarbituric acid reactive substances (TBARS) and higher monounsaturated fatty acids (MUFA) in egg yolk of quail at cold stress condition (1 to 17 °C). Interestingly, in this experiment, dose of nanoencapsulated curcumin (10 mg/kg) was three time lesser than free curcumin (30 mg/kg) which showed potential effect using nanotechnological tool. 

### 2.3. Dietary Turmeric and Curcumin in Rabbit Nutrition

Rabbit has a great importance like poultry as well as a high delicacy as a human food. However, rabbits are also very much prone to coccidiosis which is the most common digestive disease in rabbits. Due to coccidiosis, huge intestinal damage can occur in rabbits which may cause reduced body weight and nutrient-absorption capacity. As an endoparasite, *Eimeria* spp. is the most prevalent in the digestive system of farm rabbits [30]. There are few research works which have been conducted on dietary turmeric or curcumin in rabbit ailments [30,110]. Cervantes-Valencia et al. [30], for the first time, reported on the use of dietary curcumin as a natural anticoccidial alternative in adult rabbits. The results showed the positive effect of aqueous extract of curcumin on the excretion of oocysts of *Eimeria* spp. in New Zealand white rabbits such as reducing the number of oocysts and their concentration of eggs per gram of feces at the dose of 40 mg curcumin /kg body weight. However, Basavaraj et al. [110] found no beneficial effects of dietary turmeric rhizome powder (TRP) either at 0.15% or at 0.30% in the diets on blood biochemical and meat characteristics of broiler rabbits reared under summer stress conditions. 

### 2.4. Dietary Turmeric and Curcumin in Fish Nutrition

Aquaculture is considered as one of the fastest growing food producing sectors world-wide, serving a vital role in mitigating the problem of animal-protein demand, especially in the third-world countries [75]. In comparison to farm animals or poultry, fish is a better source of high quality protein, micronutrients (vitamins and minerals) and essential fatty acids, especially polyunsaturated fatty acids (linoleic acid, LA; α-linolenic acid, ALA; eicosapentaenoic acid, EPA; docosahexaenoic acid, DHA; and arachidonic acid, ARA) [72,111]. However, due to the rapid horizontal and vertical expansion of aquaculture as well as a land shortage problem, most of aquaculture is practiced under intensive and stressful farming system which causes emerging and infectious disease outbreaks [112]. In this regard, fish farmers mostly use antibiotics and chemicals to enhance the fish health status and productivity. However, unauthorized and extensive use of antibiotics in aquaculture may lead to the emergence of antibiotic-resistant bacteria which results in environmental hazards, bacteriological changes and accumulation of antibiotic residuals in fish tissues that have public health concerns [33]. To overcome the problem of unethical use of antibiotics in aquaculture, many researchers have proposed the use of herbal medicines, especially turmeric or curcumin, as antibiotic replacers in the aquaculture industry (Table 4). In a very recent report, Rajabiesterabadi [113] postulated that dietary turmeric at 10 mg/kg could ameliorate the harmful effect of copper in terms of enhancing the antioxidant parameters such as superoxide dismutase (SOD), lysozyme, catalase (CAT), glutathione peroxidase (GSH-Px) and bactericidal activity, as well as decreasing liver cytokine’s gene expressions such as tumor necrosis factor-alpha (TNF-α) and interleukins like IL1-b and IL10 in common carp. Dietary curcumin (2%) can also enhance the growth and hematological values, and antioxidant as well as immune responses in rainbow trout challenged with *Aeromonas salmonicida* subsp. *achromogenes* [114]. Baldissera et al. [63] reported that a 100% disease resistance against *Streptococcus agalactiae* in silver catfish with high appetite can be achieved when fish were offered dietary curcumin (150 mg/kg). Tilapia is one of the most cultured fish species in the world. However, there is also disease problems and health issues in tilapia aquaculture. Mahmud et al. [64] demonstrated that dietary curcumin supplementation (50 or 100 mg/kg diet) can improve the growth (increased final weight, daily weight gain, specific growth rate), feed utilization (low feed conversion ratio, high protein efficiency ratio), antioxidant status (high catalase, and low GSH and MDA activities), immune responses (increased lysozyme activity, total immunoglobulins like IgG and IgM levels) as well as disease resistance in tilapia challenged with *A. hydrophila*. Likewise, some other studies reported that dietary curcumin supplementation in percentage inclusion (0.5%, 1% or 2%) or amount inclusion in the diet (100–200 mg/kg) could enhance the growth, feed efficiency, hematology, glycogenesis, serum total protein and bactericidal activity, liver heat shock protein and disease resistance against *Vibrio alginolyticus* in Nile tilapia [32,115,116]. Furthermore, El-Barbary et al. [117,118] postulated that tilapia fish with intraperitoneal injection of AFB1 (6 mg/kg body weight) can be ameliorated by dietary garlic which showed better performance than curcumin, however, the combination of garlic and curcumin (10 mg/kg) had more benefits than high concentrations (20 mg/kg) of garlic or curcumin against aflatoxicosis. In case of freshwater climbing perch fish (*Anabas testudineus*), Manju et al. [119,120,121] explained that dietary curcumin (0.5% or 1%) or curcumin analogue as salicylcurcumin (0.5%) have protective effects against lipid peroxidation and DNA damage as well as increase the growth, survival rate and disease resistance in *A. testudineus*. Dietary curcumin can enhance the liver health status in fish. In a study, curcumin supplementation (0.5% and 1%) showed the hepatoprotective effects in Jian carp on carbon tetrachloride (CCl_4_)-induced liver damage (low aspartate transaminase (AST), alanine transaminase (ALT), hepatocyte degeneration, MDA in liver) in terms of enhanced antioxidative activities (SOD, antioxidant capacity, GSH in liver) and inhibiting related cytokine expressions (TNF-α, IL-1β) via the NF-kB signaling pathway [122]. Jiang et al. [123] reported that crucian carp offered with dietary curcumin (5 mg/kg) could improve the growth (increase final weight, feed efficiency, hepatopancreas weight), digestibility (high trypsin and lipase activities), gastrointestinal (GIT) antioxidant activities (high SOD, CAT, glutathione reductase (GR), GSH, GSH-Px, glutathione-*S*-transferase (GST) activities) and upregulation of GIT-related mRNA gene expressions (*trypsin, lipase, Na^+^, K^+^-ATPase (NKA), alkaline phosphatase (AKP), gamma-glutamyl transpeptidase (γGT), creatine kinase (CK), SOD1, CAT, GSH-Px, GST,* and *GR* genes). To date, there has been no research work conducted on curcumin nanoparticles in fish nutrition and most of the studies dealt with the extremely high dosage of curcumin in fish which seemed to be effective. However, for the sense of cost-effective and more scientific usage, we need to minimize the amount of curcumin in fish feed as an alternative to antibiotics. Therefore, it is imperative to find out correct nanoformulations of curcumin in aquafeeds to produce disease-free fish and a higher production of the aquaculture species. 

## 3. Curcumin Nanospheres in Monogastric Farm Animal, Poultry and Fish Nutrition

Natural products especially from plant sources always have a significant and promising role in pharmaceutics discovery in terms of therapeutic effects and biological properties. Curcumin as a natural therapeutic agent consists of two symmetric o-methoxy phenolic groups attached to α and β -unsaturated β–diketone which have potential as photophysical and photochemical properties [124]. 

**Figure 2:**
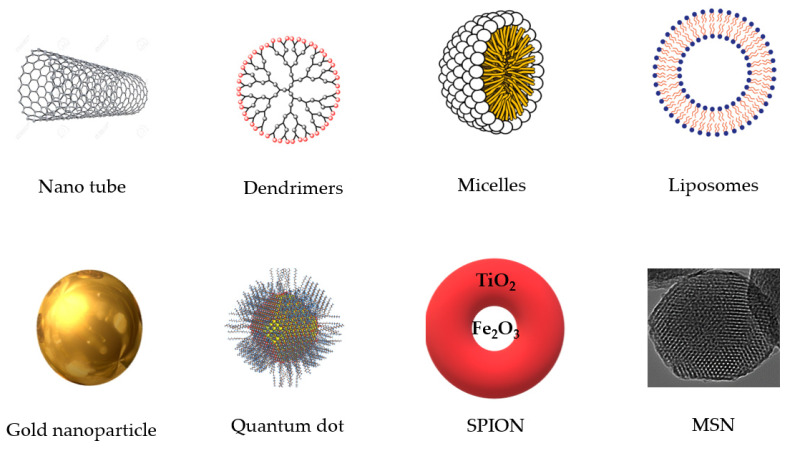
Eight different types of important nanocarriers. SPION: super paramagnetic iron oxide nanoparticles; MSN: mesoporous silica nanoparticles.

Priyadarsini [58] reported that β–diketone groups can chelate ions with formation of curcumin-metal complexes and reduce metal toxicity in living organisms. However, due to the poor solubility, poor pharmacokinetic profile and lack of proper bioavailability problems, it is needed to improve and find out a more efficient way to deliver the curcumin nanoparticles in a site-specific manner with highest bioavailability. In this regard, nanotechnological incorporation of curcumin using different nanocarriers may enhance efficacy and ensure a targeted and sustained release of curcumin [125]. Moreover, curcumin has great potential as natural pigment which may increase the feed intake in animals and due to its antioxidant properties it can produce quality and healthy animals for human consumption, which is particularly important [126]. Eight types of nanocarriers have been proposed which can be used in nanobiotechnology as smart drug delivery systems (SDDSs) (Figure 2): carbon nanotubes, dendrimers, micelles, liposomes, super paramagnetic iron oxide nanoparticles (SPION), gold nanoparticles, quantum dot and mesoporous silica nanoparticles (MSN) [127]. In vitro and in vivo studies recommend that nanocurcumin loaded with a nanocarrier has more health-span-promoting factors than traditional or native curcumin where water solubility and bioavailability of curcumin can be significantly improved through nanoencapsulation with lipid, polymeric nanoparticles, nanogels and dendrimers, and conjugating to metal oxide nanoparticles [125]. 

The effectiveness of nanodelivery systems depend on the mode of loading and release of pharmaceutic agents, long life-span as well as high therapeutic efficiency with minimum side-effects [48]. Recently, our research group developed a nanosphere loaded with curcumin which indicates curcumin nanosphere is a functional agent that can manipulate pathogenic and Gram-negative bacterium, *Vibrio vulnificus* in GIT cell deaths [128]. This concept can be further utilized in gastro-enteritis diseases in animals to reduce the overwhelming dependence on antibiotics. In addition, Suwannateep et al. [129] reported that mucoadhesive curcumin nanospheres showed 48–49% of curcumin loading and easy adherence to stomach mucosa as well as release of curcumin in blood circulation of mice model. The application of curcumin nanoparticles with a nanocarrier is not seen yet in animal nutrition or nutritional diseases, as most of the works are related to therapeutic application in human diseases, especially in Alzheimer’s disease in which curcumin pigments exhibit unique charge and bonding characteristics that facilitate penetration into the blood–brain barrier and polymerization of β-amyloid in terms of anti-Alzheimer activity [130,131], neurodegenerative disease in central nervous system or skin cancers in the form of polymer-curcumin conjugate micelles, eucalyptol microemulsions, selenium nanoparticles encapsulated PLGA nanospheres with curcumin, nanosuspension-based curcumin etc. [132,133,134,135,136]. Dende et al. [137] reported that nanocurcumin is superior to native curcumin in preventing degenerative changes in terms of cerebral malaria in mice. The experimental study showed that a nanoformulated curcumin (PLGA-curcumin) has better therapeutic index than native curcumin in preventing the onset of neurological symptoms and delaying the death of mice in experimental cerebral malaria. The result of the experiment revealed that a single oral dose of 5 mg PLGA-curcumin containing 350μg of curcumin resulted in 3- to 4-fold higher concentration and prolonged presence of curcumin in the brain than that obtained with 5mg of native curcumin, indicating better bioavailability and more effective nature of PLGA-curcumin. Recently, Baldissera and his group [71,138,139] reported the potential effect of dietary nerolidol nanospheres at 0.5 or 1.0 mL/kg diet in Nile tilapia. The results demonstrated that nerolidol nanospheres supplementation improved the growth, survival, antioxidant activities and fillet fatty acids profile and attenuated/reduced hepatic cellular damage, impaired bioenergetics, microbial contents and neuronal damage as well as disease resistance against the pathogenic Gram-negative bacterium, *Streptococcus agalactiae* in Nile tilapia. For the first time, the studies showed the benefits of nanobiotechnology in terms of fish health and meat quality as well as the field of animal nutrition. In another study, the same group of researchers have shown that the dietary curcumin-loaded nanocapsule, Eudragit L-100 polymer, enhanced the anti-inflammatory and antioxidant capacity in dairy sheep, where the doses were 10 times lower than the free curcumin [69]. There was no evidence to report the effects of nanospheres or curcumin nanosphere in monogastric farm animal or poultry nutrition. 

## 4. Conclusions and Future Outlook 

During the last decades, people have witnessed the immense popularity of nanotechnology and the potential improvement of phytogenic drugs in terms of their encapsulation to protect from enzymatic degradation, increase solubility, minimize toxicity, act on target basis with prolonged and controlled release, change the mode of pharmacokinetics and the pharmacodynamics of plant-based pharmaceutics and their overall application for mankind. Curcumin and its derivatives have potential with antioxidant, anti-inflammatory, and antimicrobial actions in applied animal science. It has safe, non-toxic and inexpensive but outstanding functional properties which make it more attractive to researchers in comparison to other phytogenic compounds. However, research works are still going on as it has some limitations due to its lipophilic and hydrophobic nature. So, there will be a great scope to use nanotechnological tools for the advancement of curcumin research in animal science. Furthermore, even though curcumin nanoparticles decrease the doses of curcumin, the nanocurcumin form may be toxic and limited in its target delivery system. Therefore, it is imperative to study the toxicokinetics of nanocurcumin in order to minimize the nanocurcumin toxicity as well as effectively deliver the curcumin in target organs of animals. From the perspective of animal nutritional biotechnology, curcumin can be more efficiently utilized using different nanocarriers especially in nanospheric form which may help to ensure effective and target-based controlled release of curcumin in different animal organs. These approaches will be helpful to improve the growth and reproductive health status, immune responses and disease resistance in terms of understanding the digestion, metabolism, immune and excretion pathways of monogastric farm animals, poultry and fishes in a more scientific way as well as to replace antibiotics on farm levels.

## Figures and Tables

**Table 1:** Effects of dietary turmeric and curcumin in swine nutrition.

Types	Animal Category and Duration of Exposure	Diet Preparation and Experimental Design	Experimental Findings	Source
Turmeric	50- to 60 -days-old weaned, crossbred pigs (Large white × Landrace × Duroc), and duration was 85–90 days of age for Experiment 1 and 175 to 185 days of age for Experiment 2.	Total 48 nursery pigs (~15 kg) with four treatments fed 0.00%, 0.05%, 0.10% and 0.20% turmeric in feed. Experiment 1, visceral organ and epithelial cell morphology on villus was observed by SEM at 30 kg BW. Experiment 2, blood was collected at 40 and 80 kg BW, growth compared at 90 kg BW	↔ Growth, carcass and visceral organ weight did not differ. ↑ nutrient digestibility at 0.10%, ↓ hematocrit at 0.10% turmeric, ↑ WBC at 0.10% and 0.20%, ↑ cell mitosis in jejunum and ileum at 0.10% and 0.20% diets	[81]
	21-days-old weaned piglets (G-Performer × Fertilium 25)/11 days of trial	Total 64 weaned piglets (~6 kg) in 2 × 4 factorial design with or without F-18 *Escherichia coli* challenged; four diets were basal or 10 ppm of capsicum oleoresin, garlic botanical, or turmeric oleoresin added	↑ average daily weight gain, ↓ ileal macrophages, diarrhea score and frequency of diarrhea, ↔ growth in diets with plant extract and challenged, F-18 *E. coli*	[82]
	21-days-old weaned piglets (G-Performer × Fertilium 25)/9 days of trial	Total 64 weaned piglets (~6 kg) divided into 4 treatments; diets were: a nursery basal diet, basal diet supplemented with 10 mg/kg of capsicum oleoresin, garlic botanical, or turmeric oleoresin	↑ expression of immunity-related genes in ileal mucosa of pigs treated with plant extracts which enhanced intestinal health status and immunity in pigs	[83]
Curcumin	21-days-old weaned piglets (Duroc × Landrace × Yorkshire)/21 days of trial	Total 50 weaned piglets (~6 kg) divided into 5 dietary treatments: control (basal diet), and the basal diet supplemented with 50 mg/kg quinocetone, or 200 mg/kg, 300 mg/kg or 400 mg/kg curcumin	↓ FCR, crypt depth, IELs, mRNA levels of IL-1β and TLR4, and TNF-α as well as ↑ villus height, goblet cells, mRNA level of IL-10 in pigs fed with 50 mg/kg quinocetone, or 300 or 400 mg/kg curcumin	[31]
	26-days-old weaned piglets (Landrace × Yorkshire)/24 days of trial	Total 40 weaned piglets (~6 kg) divided into 4 dietary treatments: NBW with control diet, NC (NBW with 400 mg/kg curcumin in control diet), IUGR with control diet, and IC (IUGR with 400 mg/kg curcumin in control diet)	↓ growth and ↑ serum pro-inflammatory cytokines, insulin resistance, hepatic lipid contents in pigs with IUGR and vice-versa in curcumin fed piglets; ↑ hepatic insulin-signaling and lopogenic pathway by IUGR but positive attenuation by IC	[68]
	26-days-old weaned female crossbred piglets (Duroc × Landrace × Large White)/26 to 115 days of age	Total 24 weaned piglets divided into 3 dietary treatments: IUGR group, NBW group, and IUGR + CUR group, which were fed diets containing 0 mg/kg (NBW), 0 mg/kg (IUGR) and 200 mg/kg (IUGR + CUR) curcumin	↑ SOD, ↓ MDA in jejunum of IUGR pigs fed CUR diet; ↑ *Nrf2, GCLC, SOD1, GCLM,* and *NQO1* mRNA gene expressions in IUGR+CUR diet; ↓ *TNFα, IL-6, IFNγ, caspase3, bax, bcl2, hsp70* ↑ *IL-2, ocln* expression in IUGR + CUR	[69]
	29-days-old weaned piglets (Large white × Landrace × Duroc)/20 days of trial	Total 192 weaned piglets (~6 kg) divided into 4 dietary levels: diets with or without (as-fed basis) quillaja saponin (750 mg/kg during week 1, 300 mg/kg during weeks 2 to 3) and with or without dietary curcumin (200 mg/kg)	↔ Growth in all treatments; ↓ ADFI, FE in quillaja saponin fed diets (days 15–20); ↑ IgG and CRP in saponin fed pigs; ↔ villus and crypt values in small intestine of all treatments;	[84]
	28-days-old weaned piglets (Duroc × Large white × Landrace)/28 days of trial	Total 180 (90 ♂ + 90 ♀) weaned piglets (~8 kg) divided into 6 dietary treatments: control diet (CON group) or supplemented diet (300 mg/kg of antibiotics, ANT group; 300 mg/kg of RES and CUR, respectively, HRC group; 100 mg/kg of RES and CUR, respectively, LRC group; 300 mg/kg of RES; 300 mg/kg CUR)	↓ *IL-1β, TNF-α* expressions and down-regulated TLR4 signaling pathway, ↑ secretion of *IgA*, *IgG* in RES + CUR or HRC fed diet in weaned piglets	[85]
	60-days-old male crossbred piglets (Pietrain × Erhualian)/21 days of trial	Total 18 piglets (~16 kg) divided into 3 dietary treatments: control (not treated with curcumin and not subjected to transport (CON), transport control (not treated with curcumin and subjected to 2 h transport together with the curcumin treated group, T-CON), and curcumin (treated with curcumin (8 mg/kg, p.o.) for 21 days and subjected to 2 h of transport thereafter, CUR) groups	↑ serum cortisol, hippocampal NO content, ↓ BDNF expression in pigs not treated with CUR; ↓ stress response, cNOS, total NOS, iNOS in CUR treated pigs;	[86]

Abbreviations: ↑ = increase; ↓ = decrease; ↔ = no change; CUR = curcumin; SEM = scanning electron microscope; RES = resveratrol; IELs = intraepithelial lymphocytes; IL-1β = interleukin 1β; TNF-α = tumor necrosis factor-α; cNOS = constitutive nitric oxide synthase; Inos = inducible NOS; NBW = new birth weight; IUGR = intrauterine growth retardation; Nrf2 = NF-E2-related factor 2; GCLC = glutamate-cysteine ligase catalytic subunit; SOD1 = superoxide dismutase 1; GCLM = glutamate-cysteine ligase modifier subunit; NQO1 = NAD(P)H quinone dehydrogenase 1; TNFα = tumor necrosis factor-α; IL-6 = interleukin-6; IFNγ = interferon gamma; IL-2 = interleukin-2; caspase3 = cysteinyl aspartate specific proteinase 3; bax = BCL2-associated X protein; bcl2 = B-cellCLL/lymphoma 2; hsp70 = heat-shock protein 70.

**Table 2:** Effects of dietary turmeric, curcumin and nanocurcumin in poultry nutrition.

Types	Animal Category and Duration of Exposure	Diet Preparation and Experimental Design	Experimental Findings	Source
Turmeric	1-day-old broiler chickens (Ross/Ross)/20-days of trial	Total 45 chickens divided into three groups; diets are: control diet, control diet supplemented with 4 mg capsicum oleoresin and 4 mg turmeric oleoresin/kg diet (XT) and uninfected or orally challenged with *Eimeria maxima* oocysts at 14 days and *Clostridium perfringens* at 18 days of age	↑ BW, ↓ gut lesion score, ↓ serum α-toxin, intestinal *IL-8*, *LITAF*, *IL-17A* and *IL-17F* mRNA levels, ↑ cytokine /chemokine in splenocytes in the XT-group compared with the birds fed the control diet	[87]
	1-day-old broiler chickens (Ross)/ 6-weeks trial	Total 240 chicks allotted into 4 groups; diets are: basal, turmeric powder (TP) supplemented at 5, 7, 9 g/kg of basal	↑ BW, liver and gizzard index, ↓ serum Cho, TG at 7 g/kg TP compared with basal diet	[88]
	288-days-old broiler chicks (Raja II)/ 42-days of trial	Total 288 broilers; basal diet with neem (8 g/kg), turmeric (2 g/kg) and vitamin E (0.2 g/kg) individually and in combination to form 8 test diet groups	↑ hematological parameters in neem fed diet; ↓ Hb in turmeric fed diet; ↑ PCV in neem, turmeric and vit E fed than basal diet	[89]
	1-day-old broiler chickens (Ross 308)/ 42-days of trial	Total 288 broilers; control diet with turmeric powder, TP at 10, 12, 14, 16, 18 and 20 g/kg, and vaccinated (positive control) or unvaccinated (negative control) to form 8 test diets	↓ BW and DFI in 20 g/kg TP fed, ↔ NDV or IBV in chicks fed TP diets compared with positive control diet; TP did not improve growth and immunity of chicks	[90]
	1-day-old Wenchang broiler chickens/ 12-weeks of trial	Total 300 broilers; basal diet with turmeric rhizome extract, TRE at 0, 100, 200 and 300 mg/kg to from 4 diets	↑ growth performance, antioxidant activity, breast muscle ↓ abdominal fat in TRE fed diets	[91]
	1-day-old Ross male chicks/ 42-days of trial	Total 625 chicks divided into 5 dietary treatment groups: thermoneutral control (TN-CON), heat stress (HS-CON), heat stressed supplemented with betaine (HS-Bet), HS with TRP (HS-TRP), and HS-BT (fed Bet and TRP)	↑ heterophil, total and IgM antibody in HS-TRP and HS-BT than HS-CON group; ↓ MDA in supplemented group than HS diet; ↑ GPx, SOD supplemented group than to TN and HS-CON groups	[92]
	52-weeks-old Hisex laying hens/8-w trial	Total 150 laying hens; control diet with turmeric powder, TP at 0, 2 and 4% to form three dietary treatment groups	↔ BW, egg weight or production at 4% TP diet; 2% TP had little effect on production, egg quality	[93]
	37-weeks-old Leghorn laying hens/ 70-days of trial	Total 160 laying hens assigned to 8 diets in 2 × 4 factorial design at 2 levels of AME (11.51 and 12.35 MJ/kg) and 4 levels of CRP (0, 2, 4 and 6 g/kg)	↑ egg shell thickness and hardness but ↓ yolk cholesterol as ↑ CRP; ↓ TG, ALT, AST and ↑ villus length, goblet cells at 2 g/kg CRP of diet	[94]
	1-week-old Japanese quails/4-weeks trial	Total 150 quails allotted to 5 diets: FM -CON (T1), SBM-CON (T2), SBM with 0.5% TP (T3), SBM with 0.5% TP and 0.1% phytase (T4), SBM with 0.5% TP and 0.1% panzyme (T5)	↑ BW, FE, total protein, albumin, SOD, CAT, GSH, GSH-Px but ↓ MDA at T3 to T5 diets compared with control diets	[95]
	1st stage: 15-days-old Japanese quails/1-month trial 2nd stage: 1-month-old Japanese quails/3-month trial	1st stage: 45 female quails divided into 3 groups like control (P0), control with 54 mg (P1) and 108 mg (P2) of TP/quail/day; 2nd stage: 3 groups like K0: offspring from parents of P0, K1: offspring from parents of P1 and K1: offspring from parents of P2 diets	After 3 months or 2nd stage: ↑ vitellogenin, HDL, vit-B12, vit-A, white egg protein, LA, ARA but ↓ Cho, LDL, total fat in eggs; ↑ carcass weight, SGPT, Cho, TG in serum, Cho in liver, but ↓ LDL and SGOT serum in female offspring	[96]
Curcumin	1-day-old chicks/ 42-days of trial	Total 180 chicks in three diet groups: control diet, control diet with lipophilic turmeric extract containing curcumin and turmerones, TF-36 at 0.5% and 1% to from 3 treatment groups	↑ BW and antioxidant activity, ↓ lipid peroxidation, ↔ meat color, serum creatinine, total protein, liver enzyme activity in 1% TF-36 fed diet than control	[11]
	1-day-old broiler (Ross×Ross)/ 14-days of trial	Two diet groups- one standard diet (control) with no curcumin and another is control with 35 mg/kg of a freeze dried *Curcuma longa* extract; At 14 day posthatch, noninfected or infected by oral gavage with 2.0 × 10^4^ oocysts of *Eimeria maxima* or *E. tenella*	↑ BW and ↓ fecal oocyst, gut lesion as well as ↑ serum antibodies, cellular immunity, in *C. longa* fed diets; dietary *C. longa* showed coccidiosis resistance against *Eimeria maxima* or *E. tenella*	[97]
	30-weeks-old laying hens (Hy-Line Brown)/ 21-days of trial	Total 60 laying hens in 3 diet groups: control (T0), control diet with curcumin at 30 (T30) and 50 (T50) mg/kg of curcumin, respectively	↑ TAC, specific gravity and yolk index of eggs, and ↓ yolk color, TBARS in eggs and ↓ oocysts in feces in the T30 and T50 fed diets	[14]
	1-day-old male chicks (Cobb 500)/ 44-days of trial	Total 225 male chicks in 5 diet groups: NC-negative control feed; PC-positive control; CU-with 50 mg/kg of curcumin, PHY-100 mg/kg phytogenic; and PHY + CU, a combination of both additives at 50 mg/kg (curcumin) and 100 mg/kg (phytogenic).	↑ total protein, globulin, ↓ uric acid, Cho, TG, oocysts, bacteria in PHY + CU diet; ↓ SFA and ↑ MUFA or PUFA in CU or PHY+CU fed groups; ↑ crypt/ villus ratio, intestinal health in PHY + CU fed diets	[66]
	1-day-old male broiler chicks (Ross × Ross)/ 21-days of trial	Total 180 chicks in 6 dietary groups: basal diet, basal diet with total curcuminoids (TCMN) at 444 mg/kg, basal diet with AFB1 at 1.0 mg/kg, basal diet with 74 mg/kg TCMN and 1.0 mg/kg AFB1, basal diet with 222 mg/kg TCMN and 1.0 mg/kg AFB1, basal diet with 444 mg/kg TCMN and 1.0 mg/kg AFB1	↑ BW, FE in 74 and 222 mg/kg TCMN at AFB1 diets; ↑ total protein, albumin, antioxidant function, γ-glutamyl transferase activity in 222 mg/kg TCMN at AFB1 diet	[15]
	21-days-old mixed sex slow-growing chickens (Thai indigenous crossbred)/63-days of trial	Total 480 chicks in six diet groups: basal diet with 4% tuna oil as positive control, basal diet added with curcumin removed turmeric oleoresin at 20, 40, 60 or 80 mg/kg curcuminoids (CUR20, CUR40, CUR60, CUR80, respectively) or dl-α-tocopheryl acetate at 200 ppm as negative control (E-200)	↑ FCR, breast fillet, yellowness of skin in curcuminoids fed diets; ↑ LA but ↓ DHA of breast meat in CUR20 and CUR40 fed diets; ↓ TBARS in chicken meat in CUR60 fed diet; suitable level of curcuminoids in slow-growing chickens was 60 mg/kg diet	[98]
	1-day-old Arbor Acres broiler chickens/ 42-days of trial	Total 400 chicks in four diet groups: control (CRM0), basal diet added with 100 (CRM100), 150 (CRM150) and 200 (CRM200) mg/kg curcumin	↑ BW, FE in CRM200 fed diet; ↑ APE and ↓ abdominal fat in CRM150 and CRM200 fed diets; ↑ villus length and width in intestine up to CRM200 fed diets	[99]
	1-day-old Arbor Acres broiler chickens/ 21-days of trial	Total 200 chicks in four diet groups: basal diet without carotenoid (control), basal diet added with 300 curcumin (CRM), basal diet added with 300 lutein (LTN) or with a combination (C + L) of 150 mg/kg curcumin and 150 mg/kg lutein; All chickens were challenged with *Eimeria maxima* at 21 d old	↑ redness and yellowness of fresh meat in C + L fed diet; ↓ MDA and carbonyl in CRM and C + L fed diets but ↑ sulfhydryl in C + L birds; ↑ myosin chain in carotenoid fed diets; CRM or C + L are efficient natural antioxidant to preserve meat quality and resistant against coccidiosis	[100]
	21-days-old male Arbor Acres broiler chickens/ 42-days of trial	Total 320 chicks in four diet groups: basal diet (C1), basal diet added with 50, 100 or 200 mg/kg curcumin (C2, C3, C4, respectively)	↑ redness value of meat, CAT, ABTS radical scavenging activity and ↓ drip loss at 48 h in curcumin supplemented groups	[17]
	21-days-old male Arbor Acres broiler chickens/ 20-days of trial	Total 400 chicks in five diet groups: basal diet + 22 °C (CON), 34 °C for 8 h (0900-1700) + basal diet supplemented with 0, 50, 100 or 200 mg/kg curcumin (HS, CMN1, CMN2, and CMN3 treatments, respectively)	↓ FCR in CMN1 & CMN2 diets; ↑ liver GSH in CMN1 & CMN2; ↑ *γ*-GCL*_m_*, GSH-Px, GST in in curcumin fed diets; ↑ Nrf2, HO-1, *γ*-GCL*c* expressions in curcumin fed diets; ↑ Cu/ZnSOD, CAT in CMN2 than the HS treatment; ↓ MDA, AST, ALT and ↑ MnSOD, mtDNA, ATP in curcumin groups;	[101,102]
	1-day-old male broiler chickens (Ross 308)/ 49-days of trial	Total 1200 chicks in four diet groups: control diet, control diet supplied with 500, 1000 and 2000 mg/kg curcumin	↓ BW, ADG, liver weight in 1000 and 2000 mg/kg CUR groups; ↓ plasma LDL and hepatic TG in 2000 mg/kg curcumin group; ↑ hepatic glycogen and hepatic lipase activities in 1000 and 2000 mg/kg curcumin groups; ↓ FAS, SREBP-1c gene expression in all curcumin group; ↑ PPARα, CPT-I expressions in 1000 and 2000 mg/kg CUR groups	[65]
	160-days-old male chicks (Ross 308)/ 42-days of trial	Total 160 chicks in four diet groups: basal diet in thermoneutral condition (23 °C), basal diet in 8 h thermal stress (34 °C), basal diet with 100 mg/kg curcumin (CR) at thermal stress (34 °C), basal diet with 1 g/kg acetylsalicylic acid (ASA) at thermal stress (34 °C)	↑ ADFI, ADG, FBW in CR added than the other diets; ↓ MDA in CR and ASA diets; ɔ PUFA, ALA, DHA in breast muscle of broiler with CR supplied diet at thermal stress (34 °C)	[103]
	1-day-old male broiler chickens/ 12-weeks of trial	Total 120 chicks in four diet groups: a 2 × 2 factorial design was used where the main factors included adding aflatoxin B1, AFB1 (< 5 vs. 100 µg/kg) and curcumin, CM (0 vs. 150 mg/kg) in a corn/soybean-based diet	↑ liver injury, ALT, AST, MDA, but ↓ albumin, total protein, CAT, GSH, GSH-Px and induced AFBO-DNA in AFB1 fed; these attributes are lowered, prevented or protected by CM added diets	[104]
	1-day-old male Arbor Acres broiler chickens/ 28-days of trial	Total 120 chicks in six diet groups: control group, curcumin alone-treated group (450 mg/kg feed), the group fed AFB1-contaminated feed (5 mg/kg feed) plus the low (150 mg), medium (300 mg) or high (450 mg) of curcumin, and the group fed AFB1-contaminated diet alone (5 mg/kg feed)	↓ liver weight and toxicity, ↑ body weight in curcumin treated groups; ↑ mRNA, protein expressions and CYP2A6 enzyme activity in AFB1-fed group; however, ↓ mRNA, protein expressions and CYP2A6 enzyme activity on dose dependent manner in curcumin fed	[105]
	22-weeks-old Roman laying hens/ 21-days of trial	Total 336 laying hens in 3 diet groups: first group as a thermoneutral control (25 °C), second group at high temp (32 °C, 6 h/day), given a basal diet, third group was five treatment groups (100, 150, 200, 250, 300 mg/kg curcumin) (H1, H2, H3, H4, H5, respectively) fed a basal diet under high temp conditions (32 °C, 6 h/day)	↑ SOD at H2 and H3 fed diets; ↑ total antioxidant capacity at H2, H3 and H5 fed diets; ↑ CAT and GSH-Px at H3 diet; ↓ MDA in curcumin added diets; ↑ CAT, SOD, GSH-Px and T-AOC in liver, heart and lungs of curcumin treated groups compared with heat stressed control group	[106]
	10-days-old Japanese quails/ 42-days of trial	Total 180 birds reared at either 22 °C (thermoneutral) or 34 °C (heat stress) for 8 h/day (0900-1700) until the age of 42 days. Birds in both environments were randomly fed 1 of 3 diets: basal diet and basal diet added with 0, 200 or 400 mg of curcumin per kg of diet.	↑ BW, FI, and ↓ FE, MDA, nuclear factor, HSP70 in response to increasing supplemental curcumin level in the diets	[107]
	1-day-old White Pekin ducklings/ 21-days of trial	Total 540 mixed-sex birds in three dietary treatments: controls (fed only the basal diet), a group fed an OTA-contaminated diet (2 mg/kg feed), and a group fed the same OTA-feed plus 400 mg/kg of curcumin	↑ BW, ADG, and no enterotoxicity in curcumin fed diet compared to OTA diet; ↓ interleukin-1β, tumor necrosis factor-α, MDA, apoptotic gene expression, mt-transcription factors and ↑ GSH, jejunal mucosa, tight junction protein in curcumin fed diets than OTA diet	[16]
	1-day-old male Cherry Valley Pekin ducklings/ 21-days of trial	Total 720 male ducklings in four dietary treatments: control group were fed a basal diet and the remainder were fed the basal diet supplemented with 200, 400, or 800 mg/kg curcumin	↑ jejunal and hepatic curcumin contents with 400 and 800 mg/kg; ↑ *SOD1, CAT, GST, GPX1, HO-1, MRP6, CYP1A4*, *CYP2D17, Nrf2* transcript, *ABCB1* and ↓ *CYP1B1*, *CYP2A6* expressions in jejunal mucosa in curcumin fed diets;	[108]
Nanocurcumin	30-days-old Japanese quails/ 21-days of trial	Total 60 birds in four diet groups: control group (T0 - without CUR), free CUR (T30 - 30 mg/kg) and two doses of CUR in nanocapsules (T3 and T10 - nanocapsules containing 3 and 10 mg of curcumin /kg of feed, respectively)	↓ FCR and ↑ egg production in T30 and T10 fed diets; ↓TBARS in egg yolk and ↑ antioxidant capacity against peroxyl radicals from T30, T3, and T10 fed diets compared to T0 diet; ↓ SFA and PUFA in egg yolk of T10 fed diet; ↑ MUFA in egg yolk of T10 and T30 fed diets	[12]
	1-day-old male broiler chickens (Ross 308)/ 42-days of trial	Total 500 chicks in five diet groups divided into two identical houses: diets were (1) control; (2) and (3) Control + 200 or 400 mg/kg curcumin; (4) and (5) Control + 200 or 400 mg/kg nanocurcumin, respectively under recommended temp up to 14 days, when the temp was dropped in one house from 28.5 to 13–15 °C and maintained at this level to induce ascites until 42 days. Whereas, in the second house the temperature was maintained according to the hybrid production guidelines	↓ WG and ↑ FCR in birds reared in cold temp than the normal temp; ↑ blood pCO_2_, HTC, and ↓ pO_2_, O_2_ saturation in cold stress at 42 d of age which is alleviated by curcumin/nanocurcumin added diets; ↑ BW, villus surface in intestine and ↓ MDA, liver enzymes, caecal *E. coli* population in curcumin or nanocurcumin fed diets	[10,109]

Abbreviations: ↑ = increase; ↓ = decrease; ↔ = no change; ɔ = restore; BW = body weight; FE = feed efficiency; FCR = feed conversion ratio; SOD = superoxide dismutase; Cho = cholesterol; TG = triglycerides; FAS = fatty acid synthase; SREBP-1c = sterol regulatory element binding protein-1c; MDA = malondialdehyde; PPARα = peroxisome proliferators-activated receptor α; CPT-I = carnitine palmitoyl transferase-I; Nrf2 = NF-E2-related factor 2; HO-1 = Heme oxygenase-1; Cu/ZnSOD = Copper and zinc superoxide dismutase; CAT = Catalase; *γ*-GCL*c* = catalytic subunit of *γ*-glutamate cysteine ligase; *γ*-GCL*m* = modulatory subunit of *γ*-glutamate cysteine ligase; GSH-Px = glutathione peroxidase; AFBO = exo-AFB1-8,9-epoxide; TAC = total antioxidant capacity; TBARS = thiobarbituric acid reactive substances; AFB1 = aflatoxin B1; HSP70 = heat shock protein 70; OTA = ochratoxin A; HTC = hematocrit; pCO_2_ = partial pressure of carbon dioxide; TLR4 = Toll-like-receptor 4 mRNA; NDV = Newcastle Disease Virus; IBV = infectious bronchitis virus

**Table 3:** Effects of dietary turmeric and curcumin in rabbit nutrition.

Types	Animal Category and Duration of Exposure	Diet Preparation and Experimental Design	Experimental Findings	Source
Turmeric	84-weeks-old weaned broiler rabbits/ 8 weeks of trial	Three treatment groups: basal diet, basal diet added with turmeric rhizome powder, turmeric rhizome powder (TRP)at 0.15% or 0.30% in the diets	no beneficial effect of dietary TRP on blood biochemical and meat characteristics of broiler rabbits reared under summer stress	[110]
Curcumin	8-months-old New Zealand white rabbits/ 42 days of trial	Total 24 rabbits in four groups: control diet, control diet with 10, 25 and 40 mg aqueous extract of curcumin /kg body weight	curcumin decreased *Eimeria* spp. oocysts excretion efficiently at a dose of 40 mg/kg BW with 80.1%, 63.7% and 64.9% for days 28, 35 and 42, respectively, with reducing concentration of eggs per gram of feces with about 20.1, 15.6 and 17.8 for days 14, 21 and 35, respectively	[30]

**Table 4:** Effects of dietary turmeric and curcumin in fish nutrition.

Types	Animal Category and Duration of Exposure	Diet Preparation and Experimental Design	Experimental Findings	Source
Turmeric	Common carp, *Cyprinus carpio* fingerlings (average body weight 42 g)/3 weeks for Experiment 1 and 3 weeks for Experiment 2	Experiment 1, total 240 fingerlings in four groups: diets supplemented with 0, 5, 10 and 20 g/kg turmeric for 3 weeks, then fish were exposed to lethal 3.5 mg/L copper for 24 h; Experiment 2, total 300 fish with same dietary groups simultaneously exposed to sub-lethal 0.25 mg/L ambient copper for 3 weeks	Experiment 1, mortality was 65.3%, 41.8%, 22.7% and 20.6% according to dietary treatments; Experiment 2, ↓ plasma cortisol, glucose, MDA, ALT, AST, and ↑ plasma T_3_, T_4_, lysozyme, ACH50, bactericidal activities, SOD, CAT, GSH-Px, RBC, Hb, and ↓ *TNF-α, IL-1β,* ↑ *IL10* genes in turmeric fed at 10 g/kg diet	[113]
Curcumin	Rainbow trout (*Oncorhynchus mykiss*) juveniles (average body weight 31 g)/ 8 weeks of trial	Total 300 juveniles in four groups: control was fed with the basal diet without CUR, the remaining groups were fed with 1% (E1), 2% (E2), 4% (E3) CUR; fish were challenged by *Aeromonas salmonicida* subsp. *achromogenes*	↑ WG, SGR, survival in E2 group; ↓ FCR, RBC, Hb, Ht in CU-fed fish; ↑ SOD, CAT, GSH-Px but ↓ MDA in liver, head kidney and spleen in CU-fed fish; ↑ hematological, immunological, antioxidant activities in fish of E2 group	[114]
	Silver catfish (*Rhamdia quelen*) juveniles (average body weight 205 g)/ 14 days of trial	Total 40 fingerlings in four groups: A-uninfected and non-supplemented fish (negative control), B-uninfected fish with 150 mg/kg CUR, C- infected & non-supplemented fish (positive control), and D- infected & added fish with 150 mg/kg CUR	100% disease resistance against *Streptococcus agalactiae* and ↓ disease sign, erratic swimming, corneal opacity, skin lesions in fin and tail, ↑ appetite in fish fed the CUR-supplemented diet;	[63]
	Tilapia *Oreochromis. niloticus*, juveniles (average body weight 2.5 g)/ 84 days of trial	Total 300 juveniles in five groups: supplemented with 0 (basal diet), 50, 100, 150 or 200 mg CUR/kg diet (CUR50, CUR100, CUR150, CUR200, respectively)	↑ WG, ADG, SGR, lysozyme, IgG, IgM in CUR50 group; ↑ FCR, PER, CP, CL in CUR100 and CUR150; ↑ CAT, GSH, ↓ MDA and survival challenged with *A. hydrophila* in all CUR fed	[64]
	Tilapia *O. niloticus*, fingerlings (average body weight 3 g)/ 100 days of trial	Total 180 juveniles in three groups: supplemented with 0% (basal group), 0.5%, or 1% CUR in the basal diets for juvenile tilapia	↑ growth, FE and GH, IGF-1, IGF-2 in fish fed the CUR supplemented diets; ↓ blood glucose, FI, leptin genes, ↑ glycogenesis in CUR fed diets	[32]
	Nile tilapia *O. niloticus*, fingerlings (average body weight 45 g)/30 days of trial	Total 60 fingerlings in two groups: commercial diets supplemented with 0% (control) and 2% CUR (weight/weight) at 2% body weight	↑ peroxidase, serum bactericidal activity, serum protein in CUR fed diet; 100% survivability in CUR fed diet challenged with *V. alginolyticus*	[115]
	Gift tilapia *O. niloticus*, juveniles (average body weight 13 g)/8 weeks of trial	Total 375 juveniles in five groups: basal diet was supplemented with 0 (control), 50, 100, 150 and 200 mg CUR/kg diets	↑ WG, FBW, SGR, ↓ CF, FCR, MDA in 150 mg/kg CUR fed diet; ↑ Serum total protein, liver HSP70 in fish fed 100-200 mg/kg CUR diets	[116]
	Tilapia *O. niloticus*, fingerlings (average body weight 40 g)/ 14 days of trial	Total 180 fingerlings in ten groups: T1, negative control; T2, injected IP with AFB1 at 6 mg/kg in basal diet; T3-T6 were fed with garlic (T3 and T4) and CUR (T5 and T6) at 10 and 20 g/kg diet, respectively; T7-T10 injected IP with AFB1 and fed both garlic and CUR at 10 and 20 g/kg diet	↓ HSI ↑ hepatic lesion in AFB1 group; ↑ HSI ↓ hepatic lesion in garlic + CUR fed groups at 10 mg/kg diet; ↑ *CYP1A* expression in CUR fed diet; ↓ toxicity of AFB1 in garlic fed diet	[117,118]
	*Anabas testudineus*, juveniles (average body weight 40 g)/ 8 weeks of trial	Total 72 juveniles in three groups: diets supplemented with 0% (basal group), 0.5%, or 1% CUR in 40% protein feeds	↓ lipid peroxidation, TBARS, but ↑ GSH and normal hepatocytes, hepatopancreas, melanocytes in fish fed the CUR fed diets	[119,120]
	*Anabas testudineus*, juveniles/ 60 days of trial	Salicylcurcumin (0.5%) as CUR analog was supplemented along with the basal diet	↓ TBARS, GSH, CAT, GSH-Px, ↑ SOD, protein content CUR fed fish	[121]
	Jian carp (*Cyprinus carpio var.* Jian), juveniles (average body weight 30 g)/ 60 days of trial	Juvenile carp were fed 0.1%, 0.5%, or 1.0% curcumin for 60 days, then injected IP with 30% CCl_4_ solution	↓ AST, ALT, hepatocyte degeneration, MDA ↑ SOD, antioxidant capacity, GSH in liver and ↓ *TNF-α, IL-1β* in fish fed 0.5% and 1.0% curcumin	[122]
	Crucian carp (*Carasius auratus*), juveniles (average body weight 76 g)/ 105-days of trial	Total 585 juveniles in three groups: diets supplemented with 0 (basal group), 1, or 5 g/kg CUR	↑ FW, PWG, FE, in 5 g/kg CUR diet; ↑ hepatopancreas weight, protein content, trypsin and lipase activities, AKP, γ-GT, CK activities in intestine, but in ↓ MDA, protein carbonyl content in 5 g/kg CUR diet; ↑ SOD, CAT, GR, GSH, GSH-Px, GST in intestine of CUR fed diets; ↑ trypsin, lipase, NKA, AKP, γGT, CK, SOD1, CAT, GSH-Px, GST, and GR gene expressions in intestine of fish fed the 5 g/kg CUR diet	[123]

Abbreviations: ↑ = increase; ↓ = decrease; ↔ = no change; CUR = curcumin; FBW = final body weight; PWG = percentage weight gain; SGR = specific growth rate; ADG = average daily gain; FI = feed intake; FE = feed efficiency; FCR = feed conversion ratio; HSI = hepatosomatic index; CF = condition factor; PER = protein efficiency ratio; CP = crude protein; CL = crude lipid; AFB1 = aflatoxin B1; IP = injected intra peritoneal; Ig = immunoglobulin; SOD = superoxide dismutase; CAT = catalase; GSH = glutathione; GSH-Px = glutathione peroxidase; GST = glutathione-*S*-transferase; GR = glutathione reductase; MDA = malonaldehyde; AST = aspartate transaminase; ALT = alanine transaminase; RBC = red blood cells; Hb = hemoglobin; TBARS = thiobarbituric acid reactive substances; ACH50 = alternative complement hemolytic; TNF-α = tumor necrosis factor-alpha; IL1-β = interleukin 1-beta; HSP70 = heat shock protein 70; CCl_4_ = carbon tetrachloride; AKP = alkaline phosphatase; γ-GT = gamma-glutamyl transpeptidase; CK = creatine kinase; NKA = Na^+^, K^+^-ATPase;
